# Breast Cancer Incidence Trends and Projections in Northeastern Thailand

**DOI:** 10.2188/jea.JE20170045

**Published:** 2018-07-05

**Authors:** Shama Virani, Jarin Chindaprasirt, Kosin Wirasorn, Aumkhae Sookprasert, Ongart Somintara, Damnern Vachirodom, Supinda Koonmee, Jirapon Srinakarin, Supot Kamsa-ard, Krittika Suwanrungruang, Laura S. Rozek, Hutcha Sriplung, Surapon Wiangnon

**Affiliations:** 1 Epidemiology Unit, Faculty of Medicine, Prince of Songkla University, Hat Yai, Songkhla, Thailand; 2 Medical Oncology Unit, Department of Medicine, Faculty of Medicine, Khon Kaen University, Khon Kaen, Thailand; 3 Department of Surgery, Khon Kaen University, Khon Kaen, Thailand; 4 Department of Pathology, Faculty of Medicine, Khon Kaen University, Khon Kaen, Thailand; 5 Department of Radiology, Faculty of Medicine, Khon Kaen University, Khon Kaen, Thailand; 6 Cancer Unit, Srinagarind Hospital, Faculty of Medicine, Khon Kaen University, Khon Kaen, Thailand; 7 School of Public Health, University of Michigan, Ann Arbor, MI, USA; 8 Department of Pediatrics, Faculty of Medicine, Khon Kaen University, Khon Kaen, Thailand

**Keywords:** breast cancer, northeast, Thailand, epidemiology, surveillance

## Abstract

**Background:**

The northeast has the lowest incidence of breast cancer of all regions in Thailand, although national rates are increasing. The heterogeneity in subnational trends necessitates a comprehensive evaluation of breast cancer incidence trends and projections to provide evidence for future region-specific strategies that may be employed to attenuate this growing burden.

**Methods:**

Joinpoint regression and age-period-cohort modeling were used to describe trends from 1988–2012. Data was projected from three separate models to provide a range of estimates of incidence to the year 2030 by age group.

**Results:**

Age-standardized rates (ASRs) increased significantly for all women from 1995–2012 by 4.5% per year. Rates for women below age 50 increased by 5.1% per year, while women age 50 years and older increased by 6% per year from 1988–2012. Projected rates show that women age 50 years and older have the largest projected increase in ASRs by 2030 compared to younger women and all women combined.

**Conclusions:**

Breast cancer trends in Khon Kaen are presently lower than other regions but are expected to increase and become comparable to other regions by 2030, particularly for women ages 50 years and older.

## INTRODUCTION

Breast cancer is the most frequently diagnosed cancer in women, making up 25% of total cancers diagnosed in women worldwide.^1^^,^^2^ Almost half of all breast cancer incidence and approximately 60% of breast cancer mortality occurs in low- and middle-income countries, including Thailand.^1^ Breast cancer incidence has increased in Thailand over the past 20 years and currently has the highest incidence of all female cancers nationally, with an age-standardized rate (ASR) of 28.5 cases per 100,000 person-years (PY).^3^ Within Thailand, incidence rates vary geographically, illustrating the diverse lifestyles, behaviors, and risk profiles of the northern, northeastern, central, and southern regions of Thailand. Due to this heterogeneity of incidence rates, it is necessary to characterize breast cancer trends by region to determine the local burden and assess need for prevention strategies.

The northeast has the lowest incidence of breast cancer, with an ASR of 19.4 cases per 100,000 PY, compared to the northern, central, and southern regions, with ASRs of 32.4, 33.9, and 27.4 cases per 100,000 PY, respectively.^3^ Khon Kaen, a province in the northeast, has the highest incidence of breast cancer in the region, with 23.0 cases per 100,000 PY. Khon Kaen Province has been a focus for cancer researchers, largely due its high burden of liver cancer. However, with the largest incidence in the northeast, and with the overall increasing incidence of breast cancer in Thailand, determining the present and future burden from breast cancer in Khon Kaen is critical to providing evidence for prevention strategies that may be employed to attenuate this burden in the northeastern region.

Here we provide a comprehensive assessment of breast cancer incidence trends in Khon Kaen and project incidence rates into the future to describe the regional impact of breast cancer on women in northeastern Thailand.

## METHODS

### Region

**Figure 1.**
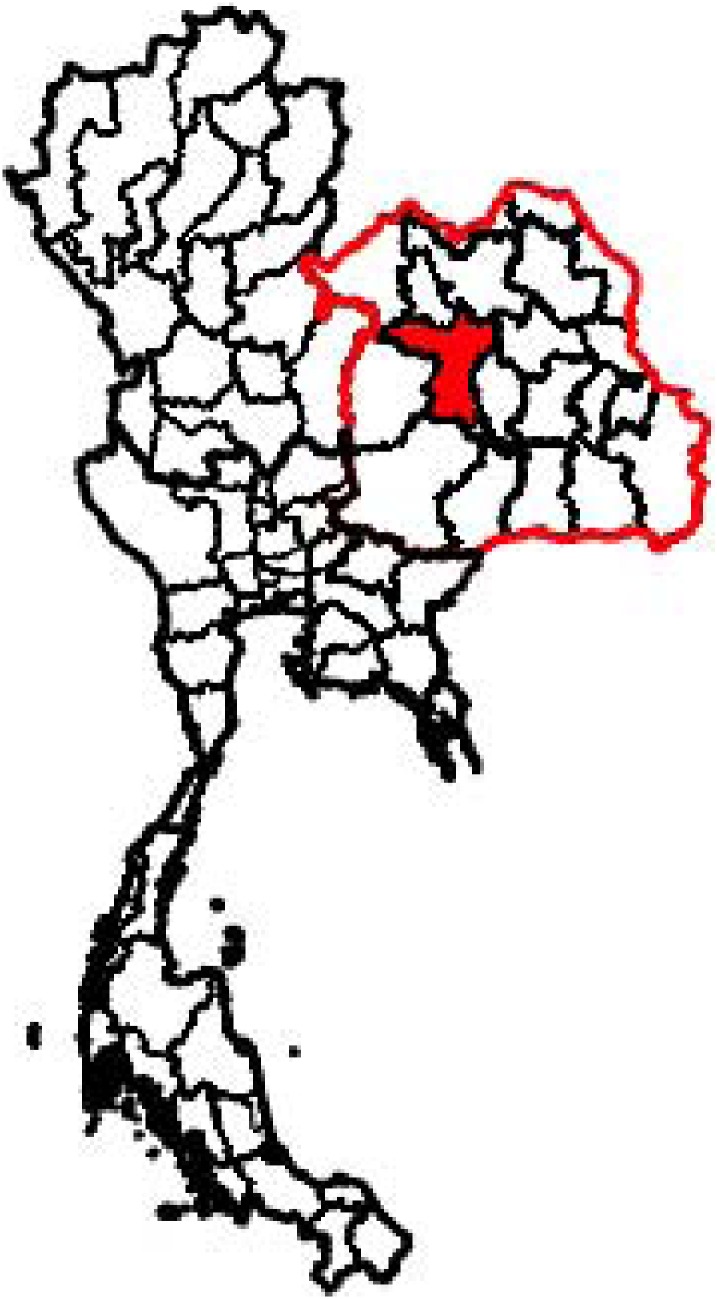
Khon Kaen is the fifth largest province in the northeastern region of Thailand

Khon Kaen, Thailand is the fifth largest northeastern province, occupying 10,886 km^2^ (Figure 1). The population of the northeastern region at the 2010 census was 18.9 million, of which 9.6 million were female.^4^

### Khon Kaen Cancer Registry

Strict data collection, registration, and maintenance protocols of this registry allow for reliable estimates of cancer incidence. As this is the only registry in the region with these standards, it is used as a basis to understand cancer incidence in the northeastern region of Thailand.

The Khon Kaen registry collects cases from 28 districts in northeastern Thailand. This registry compiles cases both actively and passively from a university hospital (Srinagarind hospital), a regional hospital (Khon Kaen provincial hospital), an army hospital, two private hospitals, and 24 community hospitals. Registrars are trained in collection protocols developed by the International Agency for Research on Cancer (IARC). The capture-recapture technique was used to monitor completeness of the cancer registry.^5^ All data are verified, checked for duplication, coded, and entered into CanReg5 software (International Association of Cancer Registries, Lyon, France).

### Data

Female breast cancer cases, including information on age and date of diagnosis, were extracted from the Khon Kaen Cancer Registry from 1988–2012 using ICD-10 codes C50.X. Data quality, in terms of percent morphologically verified and percent of death certificate only, for female breast cancer was high and increased over time (1988–1991: %MV, 76.4; %DCO, 3.1 and 2010–2012: %MV, 94.3; %DCO, 0.2).^3^^,^^6^ Population numbers, used to calculate incidence rates, were retrieved from population censuses conducted in 1990, 2000, and 2010.^4^^,^^7^^,^^8^ Intercensus populations were estimated using a log-linear function between two consecutive censuses. Population numbers beyond 2010 were estimated, and reported by the Office of the National Economic and Social Development Board.^9^

### Trend analysis

ASRs calculated for each year from 1988–2012 were standardized to the modified Segi world population.^10^^,^^11^ Observed trends were analyzed using the Joinpoint Regression Program version 4.2.0.2 (Statistical Methodology and Applications Branch, Surveillance Research Program, National Cancer Institute, Bethesda, MD, USA). Joinpoint regression identifies statistically significant trend change points (joinpoints) and the rate of change (annual percent change) in each trend segment using a Monte Carlo permutation method.^12^ Analyses were conducted for all females, and then for females younger than the age of 50 and females 50 years of age or older, to determine the differences in incidence trends above and below the mean age of menopause.^13^^,^^14^

Age-Period-Cohort (APC) regression models were used to investigate the effects of age, calendar year, and birth cohort on the incidence of breast cancer. Age-specific incidence rates were calculated for single-year age groups. The classical method was used as described previously.^15^^,^^16^ Briefly, a log-linear model with a Poisson distribution was fit to the data. To address the non-identifiability problem of the APC models, two-effects models (age-period and age-cohort) were first chosen, and the remaining effect (cohort or period) was constrained to be 0 on average with 0 slope, yielding an APC and an ACP model. The analysis of APC models was performed using the Epi package^17^ for R statistical software version 3.1.2 (R Foundation for Statistical Computing, Vienna, Austria).^18^

### Rate projection with comparative modeling

Three independent methods, joinpoint, APC, and nordpred, were used to project incidence rates to the year 2030.

#### Joinpoint

Each best-fit joinpoint model was extrapolated to the year 2030 using the intercept and slope values from the most recent significant trend. Attenuation or dampening of the linear drift was used address the concept that past trends will not continue to increase linearly. Here, we used a combination of attenuation methods from Moller et al^19^ and Mistry et al.^20^ The first 5 projected years had 0% attenuation, followed by geometric dampening at a rate of 8% each year until 2030.

#### Age-Period-Cohort

Projections for this method were based on the work of Carstenson.^21^ Briefly, linear interpolation was used to project period and cohort effects of the model. Age-specific rates were calculated for each year using the age, and projected period, and cohort effects. Rates were smoothed with averages to adjust for occasional outliers beyond 5%. ASRs were calculated using the modified Segi world population.^10^^,^^11^ The linear drift was extracted from the APC model using the Holford method, which uses the naive average over all values for the estimated effects and disregards the number of cases.^22^ Geometric dampening was applied to the linear drift as explained above for joinpoint.

#### Nordpred

The nordpred R package was used to fit an APC model with a power5 link function to observed data using 5-year interval periods and eighteen age groups. The average trend based on all observed data was extrapolated out to the year 2030. Geometric dampening, as described above, was applied to the vector of proportions of drift that were cut in each projection period. Observed and attenuated rates for each 5-year period were compiled for the total population, women younger than 50 years, and women aged 50 or older. Natural splines were fit to 5-year ASR values to obtain ASRs for each single year in each group. The numbers of projected cases were calculated from the projected ASRs, estimated population numbers and the Segi world population.

Joinpoint Regression Program v4.2.0.2 and the R-statistical software were used for trend analysis and prediction (Epi 1.1.71 and NORDPRED, R version 3.2.1).^17^^–^^19^^,^^23^ This study was approved by the Thai Ethics Committee (REC 58-013-18-1).

## RESULTS

In Khon Kaen, from 1988–2012, there were a total of 3,743 invasive breast cancer cases, with 1,814 cases in women younger than 50 years and 1,929 cases in women aged 50 years or older. Stage distributions (Table 1) across each 5-year period show incidence of localized cases increasing from 3.0% in 1988–1992 to 13.2% in 2008–2012, although this stage makes up the smallest proportion of all stages in each 5-year period. The largest proportion of cases in each 5-year period is regional, ranging from approximately 33–64%. The percent of distant and unknown cases decreased from 1988–2012.

**Table 1.** Stage distribution by age group

	All	<50 years	≥50 years
1988–1992			
Localized	3.0%	2.7%	3.4%
Regional	32.6%	30.8%	34.7%
Distant	29.5%	27.4%	32.2%
Unknown	34.8%	39.0%	29.7%
1993–1997			
Localized	5.1%	6.5%	3.2%
Regional	40.4%	41.0%	39.7%
Distant	20.2%	18.8%	22.2%
Unknown	34.2%	33.7%	34.9%
1998–2002			
Localized	4.4%	4.0%	4.8%
Regional	56.5%	57.1%	55.9%
Distant	8.3%	8.3%	8.4%
Unknown	30.7%	30.6%	30.9%
2003–2007			
Localized	7.2%	7.0%	7.3%
Regional	44.1%	42.0%	46.1%
Distant	11.4%	10.2%	12.6%
Unknown	37.3%	40.8%	34.1%
2008–2012			
Localized	13.2%	15.0%	11.8%
Regional	64.2%	63.1%	65.0%
Distant	8.4%	7.5%	9.1%
Unknown	14.2%	14.4%	14.1%

Proportions add up to 100% vertically for each period.

**Figure 2.**
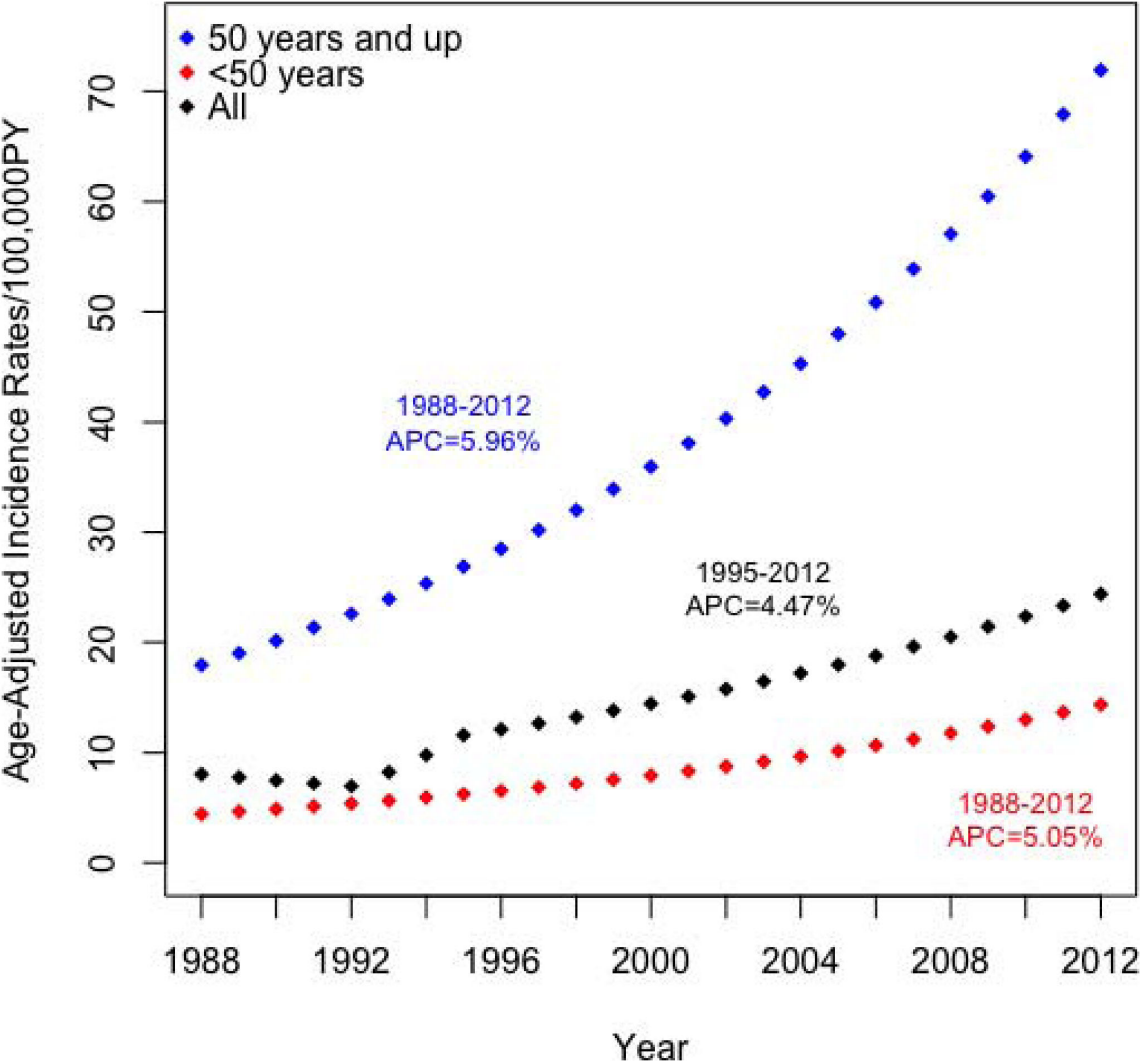
Age standardized incidence rates for all women and by age group from 1988–2012. Annual Percentage Change (APC) for each age group is shown

Trend analysis revealed that ASRs did not change significantly from 1988 to 1995, when the ASRs increased from 7.9 to 10.5 cases per 100,000 PY (Table 2 and Figure 2). However, from 1995 to 2012, ASRs increased significantly by 4.5% per year for all women, to 24.0 cases per 100,000 PY in 2012. In women aged younger than 50 years, ASRs increased significantly from 1988–2012 by 5.0% per year, although the rates remained lower than those for all women. For this group, ASRs increased from 4.0 cases per 100,000 PY in 1988 to 12.4 cases per 100,000 PY in 2012. In women aged 50 years and older, the ASR increased from 23.5 to 70.3 cases per 100,000 PY from 1988 to 2012, a significant increase of 6.0% per year.

**Table 2.** Trend analysis of significant annual percent changes by age

Age Group	Trend 1		Trend 2		Trend 3	
		
	Years	APC	Years	APC	Years	APC
All	1988–1992	−3.6%	1992–1995	18.60%	1995–2012	4.5%^*^
<50 years	1988–2012	5.1%^*^				
≥50 years	1988–2012	6.0%^*^				

APC, annual percent change.

^*^Significant at *P* < 0.05.

**Figure 3.**
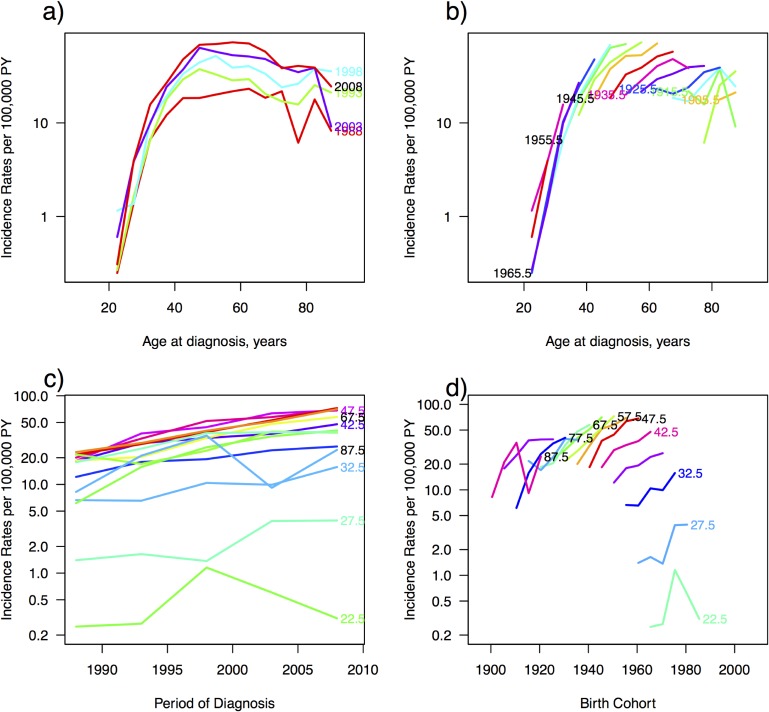
Incidence trends by age at diagnosis for a) each 5-year period of diagnosis and b) birth cohort. Incidence trends for 5-year age groups by c) period of diagnosis and d) birth cohort.

**Figure 4.**
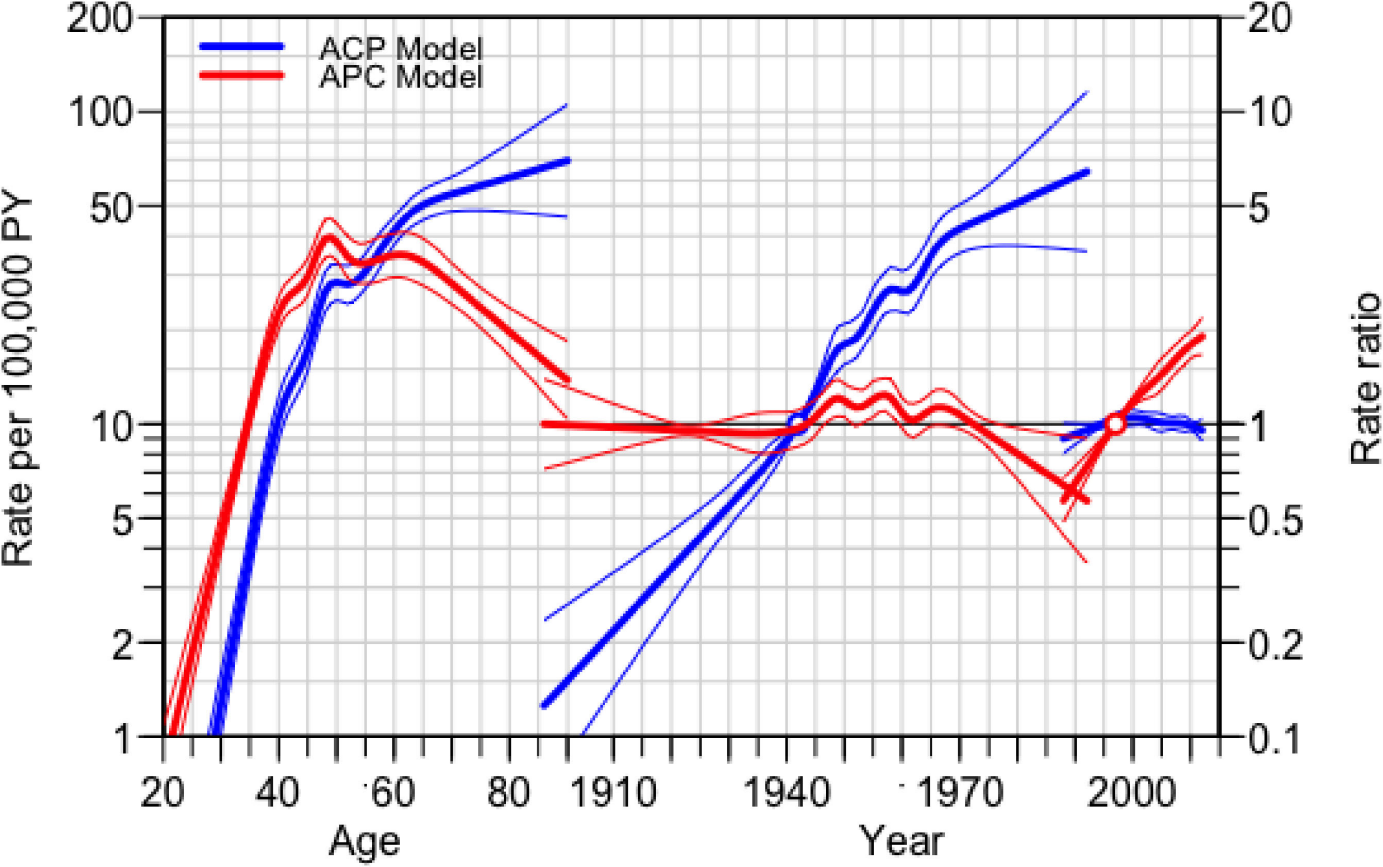
Age-Period-Cohort model (red) and Age-Cohort-Period (blue) models showing age (left), cohort (center) and period (right) effects

Incidence rates increase with age for all 5-year periods; however, the most recent period had the highest incidence rates for all age groups, while the first period had the lowest (Figure 3a). Incidence rates were highest for oldest cohorts and decreased for younger generations (Figure 3b and Figure 3d). Older woman had higher rates of breast cancer with women between the ages of 45–55 years exhibiting the highest incidence across all years of diagnosis (Figure 3c). Age-period-cohort modeling revealed significant period and cohort effects (Figure 4). The APC model (red) exhibited a peak in rates at 49 years (left) with a rate of 38.4 cases per 100,000 PY (95% CI, 33.3–44.4). The period effect has a rate ratio of 0.6 in 1988 (95% CI, 0.5–0.7) and reaches 1.9 (95% CI, 1.7–2.3) in 2012 (right). In the ACP model (blue), rates increase by age, reaching it’s first peak at age 48 (left), with a rate of 25.4 cases per 100,000 PY (95% CI, 21.9–29.4) and the continues to increase. Cohort effects (center) peak in 1958, with a rate ratio of 2.7 (95% CI, 2.3–3.2) and then continue to increase.

### Projections

**Figure 5.**
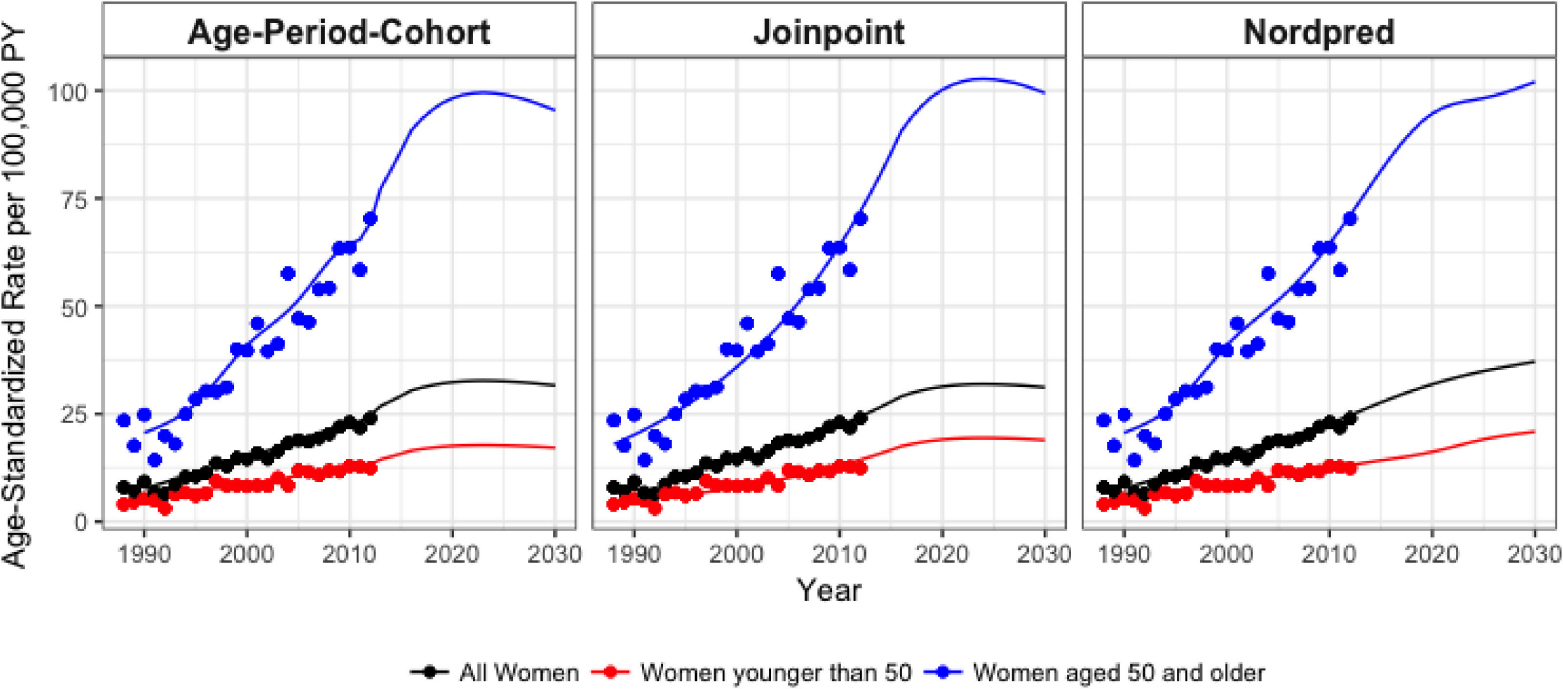
Age-standardized incidence rate projections to the year 2030 as estimated using each method. Observed values (dots) are shown from 1988–2012 while modeled and projected rates (lines) are shown from 1988–2030.

Age-adjusted incidence rates were projected using three different models—joinpoint, APC, and nordpred—to assess the future burden of breast cancer in Khon Kaen up to the year 2030. For joinpoint projections, geometric dampening was applied to projected ASRs to attenuate the linear increase over time. The joinpoint model projected incidence rates for all women to peak at 31.9 cases per 100,000 PY (492 cases) in 2023 and remain relatively stable, reaching 31.2 cases per 100,000 PY (481 cases) in 2030. Women younger than age 50 reached a maximum rate at 19.4 cases per 100,000 PY (126 cases) in 2023 before dropping to 18.9 cases per 100,000 PY (110 cases) in 2030. Rates for women aged 50 years and older peaked in 2024, with an ASR of 102.7 cases per 100,000 PY (372 cases), before dropping to 99.5 cases per 100,000 PY (371 cases) in 2030 (Figure 4 and Figure 5).

The APC projections for all women reached a maximum of 34.7 cases per 100,000 PY (466 cases) in 2022 and remained stable, ending at 33.4 cases per 100,000 PY (455 cases) in 2030. Rates for women younger than age 50 peaked in 2023 at 17.8 cases per 100,000 PY (115 cases) and ended at 17.2 cases per 100,000 PY (99 cases) in 2030. Women aged 50 years and older had rates that also peaked in 2023 at 100.5 cases per 100,000 PY (355 cases) and remained relatively stable until 2030, with an ASR of 96.2 cases per 100,000 PY (356 cases) in 2030.

**Figure 6.**
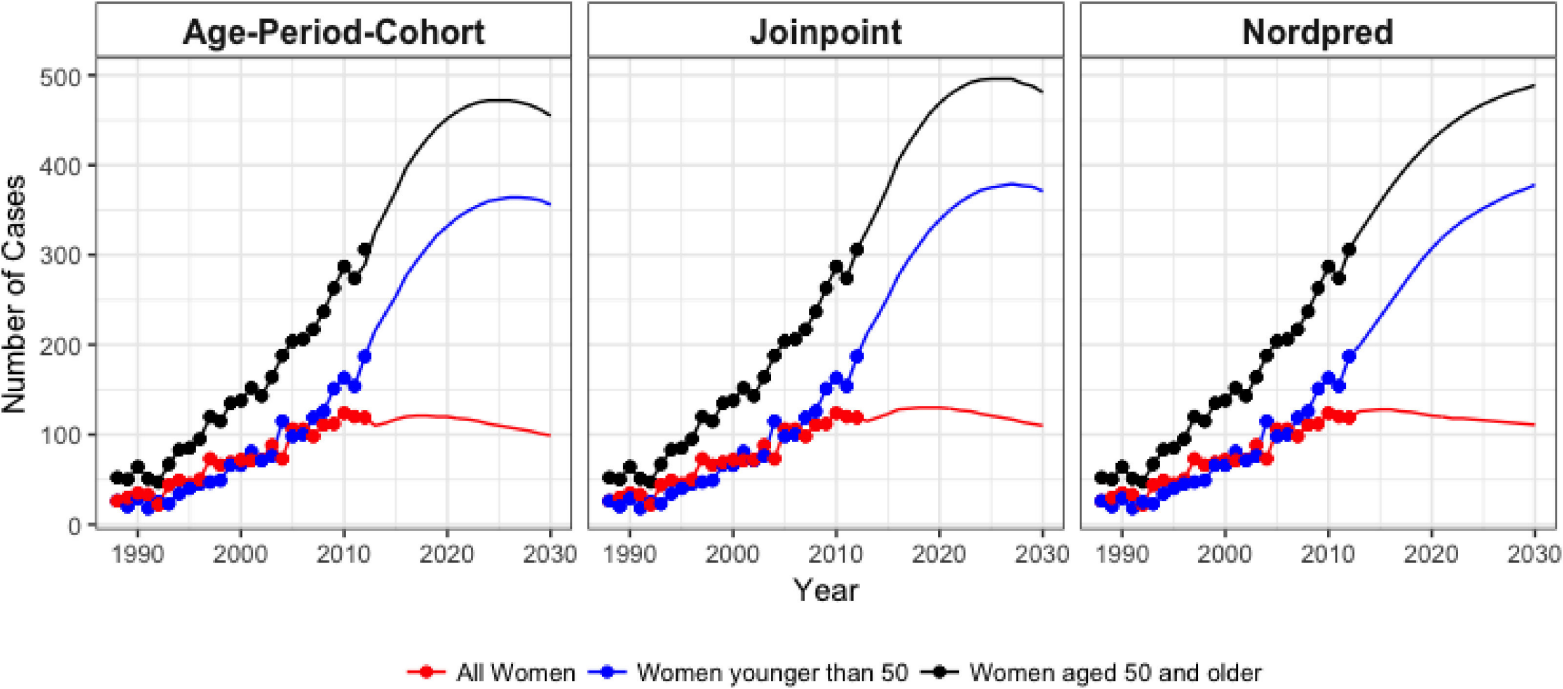
Number of cases were calculated for the observed period (dots) and extrapolated to year 2030 (lines) using each method

Rate projections for all women from the nordpred model reached 37.1 cases per 100,000 PY in 2030, while they were 20.9 cases per 100,000 PY and 102 cases per 100,000 PY for women younger than age 50 and women aged 50 years or older, respectively. Using projected population data, this translates to 489 cases, 111 cases, and 378 cases for all women, women younger than 50 years, and women aged 50 years and older in 2030 (Figure 5 and Figure 6).

## DISCUSSION

We show that breast cancer trends in Khon Kaen have increased significantly in the last 20 years and will continue to do so if no prevention measures are introduced, particularly for women ages 50 and older. These findings can be extended to the entire region, as the northeast has a relatively homogenous population.^4^

Over the years, the majority of breast tumors have been increasingly staged as localized or regional regardless of age group, particularly in the most recent period. However, the proportion of localized tumors exhibited a slowly increasing trend, whereas regionally staged tumors exhibited an increasing trend with a larger magnitude. This is likely due to delayed diagnosis, which is prominent in this region. Although the northeast has the highest rates of breast self-examinations in Thailand,^24^^,^^25^ a previous study showed that lack of hospital referrals from local healthcare providers in this region is a major contributor to delayed diagnosis.^26^ These factors have important implications for stage presentation at diagnosis and highlights the need for early detection strategies as a preventive measure and to downstage these tumors. Opportunistic mammography is available in Khon Kaen and is highly utilized, with waiting times lasting up to 1 year due to limited number of machines and radiologists.^27^^,^^28^ This might explain the increasing proportion of localized tumors over time across all ages. In 2013, the fast-track mammography program was established to reduce delays in treatment through adherence to strict timelines for diagnostic mammography, pathology reports, surgery, and treatment. The National Health Statistics Office reports 80% adherence to the timeline and provides a successful working model for potential expansion to other regions.^29^

**Figure 7.**
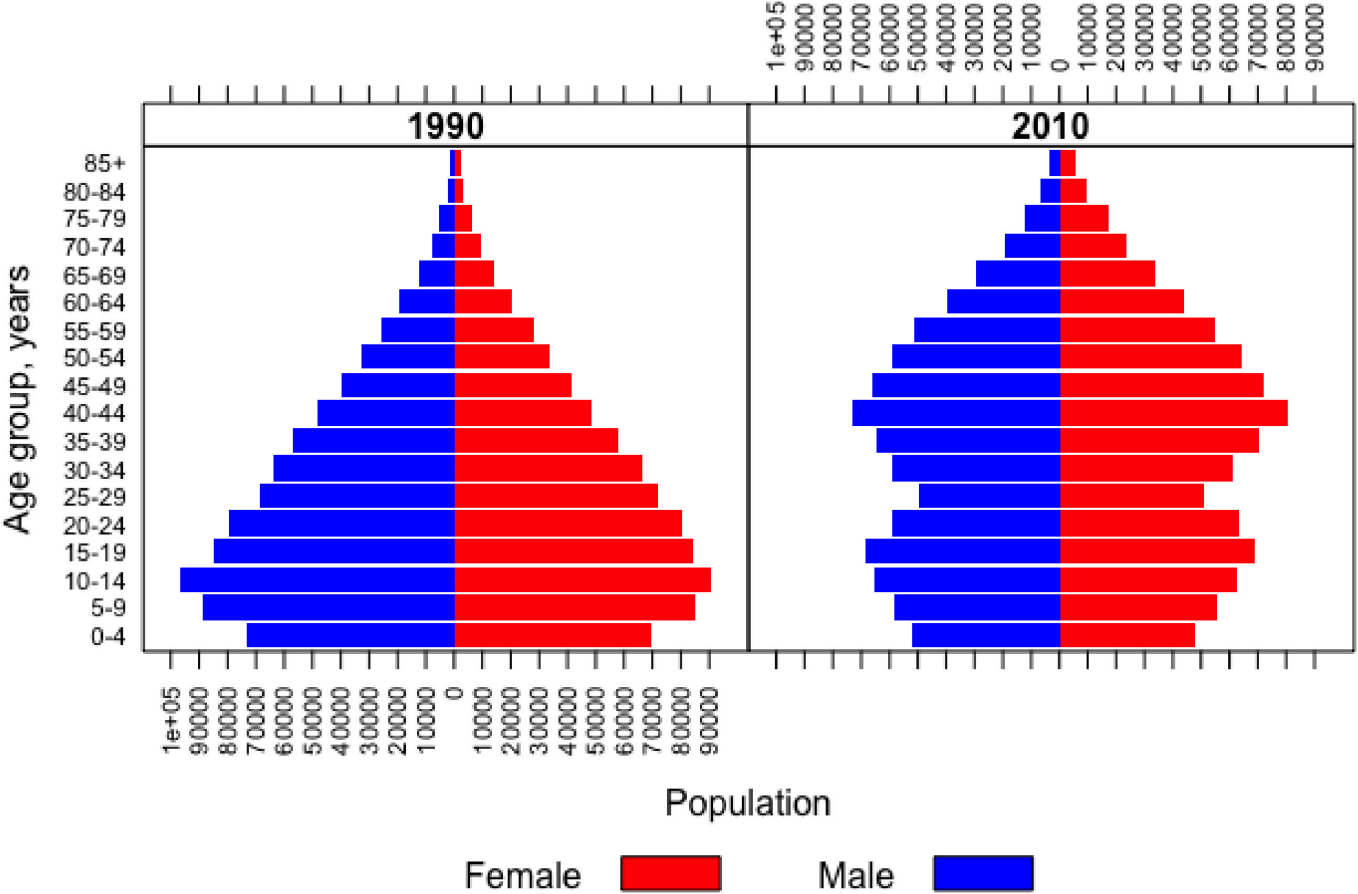
Population distributions for males and females in 1990 and 2010 by 5-year age groups

The observed ASR in 2012 Khon Kaen (24 cases per 100,000 PY) falls below the national Thai average of 28.5 and below the ASRs of other regions in Thailand. The ASR (per 100,000 PY) in the south was 27.8 in 2010, and estimates for the northern and central regions were 26.3 and 34.3, respectively.^3^^,^^15^^,^^16^^,^^30^^,^^31^ However, with an aging population increasing the proportion of postmenopausal women (Figure 7), the rising breast cancer rate in women at age 50 and older is an important contributor to the cancer burden of this region. Still, incidence for women in this age group was lower in this region in 2012 (70 cases per 100,000 PY) than in the southern (74.4 cases per 100,000 PY), central (85 cases per 100,000 PY), and northern (110 cases per 100,000 PY) regions.^3^^,^^15^^,^^16^^,^^30^^,^^31^ However, expected incidences by the year 2030 in this region are comparable to other regions. Incidence for all women in Khon Kaen is expected to reach 31–33 cases per 100,000 PY in 2030, while expected ASRs for the northern, central, and southern regions are 30–35, 29–31, and 44–45 cases per 100,000 PY, respectively. Similarly, incidence rates in Khon Kaen for women aged 50 years or older are expected to be comparable to other regions by 2030.^3^^,^^15^^,^^16^^,^^30^^,^^31^ These comparisons illustrate that, while breast cancer ASRs are currently low in the northeast, they will catch up to other regions in the future.

There may be several reasons for the lowest observed incidence in the northeast. First, the northeastern region contains 26% of Thailand’s population and 40% percent of Thailand’s poor, resulting in the highest poverty incidence by regional population and lowest per capita income in Thailand.^32^^–^^34^ Low-income populations tend to be less likely to be diagnosed with breast cancer and more likely to die from the disease.^35^ In addition, the northeast has the second shortest life expectancy in Thailand for both males and females at 69.6 and 76.4 years, respectively.^36^ Considering the proportional relationship between poverty and risk factors for infectious diseases, there may be competing causes of mortality in this region that contribute to lower rates of breast cancer.^37^ Therefore, it is possible that there is still a large burden of infectious diseases in the northeast that may account for a larger proportion of deaths than noncommunicable diseases. It may be that this region is lagging in its epidemiologic transition and breast cancer burden compared to other regions. Although it is difficult to quantify this in absolute terms, there are several indicative measures available. The northeast has the highest percentages of low birth weight infants, moderate prevalence of underweight and stunted children, and severe prevalence of children with no vaccinations against childhood diseases. It also has the lowest proportion of women vaccinated against tetanus.^38^ The main source of drinking water in the northeast is rain-water collection, and this region has the highest proportion of households that do not use any form of water treatment methods, such as boiling, straining, or filtering.^38^ Finally, this region has the second-highest percentage of use of unimproved sanitation facilities (0.8%), with the highest percentage being in the south (1%).^38^ All of these factors contribute to spread of infectious diseases and provide competing causes of mortality in this region.

Second, the prevalences of risk factors for breast cancer are lower in the northeast compared to other regions. Despite the fact that fertility rates have decreased overall in Thailand, the northeast has the highest fertility rate of 2.2 births per woman, compared to all other regions in Thailand.^38^^,^^39^ This region also has high rates of exclusive breastfeeding for the first six months,^38^ the lowest prevalence of overweight or obese women,^40^ and the lowest rates of current and daily smokers^41^ of all regions. In addition, although prevalence of alcohol consumption in women was higher compared to other regions, the amount of alcohol consumed was low.^42^ Higher prevalence of protective factors likely contributes to the incidence rates described here.

The rising incidence trend of breast cancer in this region indicates that breast cancer is increasing in the northeast, as expected considering worldwide trends. However, it is important to note that these trends offer information from a population-based cancer registry. The data quality of this registry has improved over time, and this fact may partially contribute to the magnitude of increases shown here. Therefore, while the trends are increasing over time, the influence of improvements in data quality on the magnitude of increase must be considered when assessing ASRs over the years.

Characterizing breast cancer trends is vital to understanding the trajectory of the disease. However, a limitation of this study is the lack of cancer subtype information, which likely plays a large role in the surveillance, treatment, and survival outcomes of this disease. As breast cancer is no longer viewed as a single disease, including subtype distribution would aid in understanding incidence trends associated with breast cancers. Future studies incorporate these types of assessments to provide a more comprehensive understanding of breast cancer in Thailand.

Given the high access to care in Thailand due to its universal health coverage system and its strong infrastructure, which provides care down to the village level through community health volunteers, there are many avenues to prevention and earlier diagnosis of breast cancer in this region that would ease the burden of this deadly disease. Directed educational programs can target the most vulnerable populations, such as women aged 50 years or older and those with known family history of disease. Prevention strategies should continue to promote self-breast examinations, breastfeeding, and other protective behaviors. This is especially important considering that, while rates are currently lower in this region than all others, they are expected to become comparable in the future. Future strategies should also focus on identifying effective prevention measures that address risk factors associated with high rates of poverty, as this will likely continue to play a role in the breast cancer burden in the future.
